# Maladaptive DNA damage responses impair CD8 T‐cell fitness after allogeneic stem cell transplantation

**DOI:** 10.1002/hem3.70525

**Published:** 2026-11-04

**Authors:** Tobias Ronny Haage, Domenica Saul, Romy Böttcher‐Loschinski, Martin Böttcher, Simon Völkl, Andreas Mackensen, Matthias Leisegang, Maurice Michel, Denise Walther, Dimitrios Mougiakakos

**Affiliations:** ^1^ Department for Hematology, Oncology, Cell‐ and Radiotherapy Otto von Guericke University Magdeburg Germany; ^2^ Department of Internal Medicine 5, Hematology and Oncology Friedrich Alexander Universität Erlangen‐Nürnberg and Universitätsklinikum Erlangen Erlangen Germany; ^3^ Department of Oncology and Pathology, Science for Life Laboratory and Center for Molecular Medicine Karolinska Institute and Karolinska Hospital Stockholm Sweden; ^4^ Magdeburg Center for Cell‐ and Immunotherapy Otto von Guericke University Magdeburg Germany

Allogeneic hematopoietic stem cell transplantation (allo‐SCT) remains the only curative option for selected hematologic malignancies. Its therapeutic efficacy critically depends on donor‐derived T‐cells mediating the graft‐versus‐leukemia effect. Early T‐cell reconstitution after allo‐SCT is driven by expansion and allo‐activation of transferred donor T‐cells, whereas long‐term immune recovery relies on thymic regeneration.^1^ However, T‐cell activation is inevitably associated with abundant production of reactive oxygen species (ROS).^2^ Although ROS are essential for T‐cell signaling and effector differentiation, excessive oxidative stress can damage cellular components, including DNA.^3^ One common form of oxidative DNA damage is 8‐hydroxy‐2′‐deoxyguanosine (8‐OHdG), which has been linked to impaired T‐cell function, exhaustion, and inferior survival after allo‐SCT. We previously demonstrated that high levels of oxidative DNA damage in reconstituting T‐cells correlate with dysfunction and increased relapse risk following transplantation.^4^ In addition, we showed that activation of 8‐oxoguanine DNA glycosylase 1 (OGG1), a key initiator of the base excision repair (BER) pathway responsible for removal of oxidative DNA lesions such as 8‐OHdG, not only enhances repair of oxidative DNA damage but also improves T‐cell functionality and protects from exhaustion.^5^ These findings suggest that DNA damage responses (DDRs) are tightly connected to T‐cell fitness after allo‐SCT. Despite evidence that allo‐reactive T‐cells activate stress‐protective programs such as nuclear factor erythroid 2‐related factor 2 (NRF2) signaling,^6^ little is known about the regulation and functional relevance of DDR pathways in donor T‐cells after transplantation. This study aimed to characterize DDR activity after allo‐SCT and identify biomarkers and therapeutically targetable mechanisms associated with effective T‐cell immunity. Intervening at potential points within DDR pathways may improve post‐transplant recovery.

Peripheral blood samples from allo‐SCT recipients (at Day +30 post‐allo‐SCT) and healthy donors (HDs) were collected after written informed consent in accordance with the Declaration of Helsinki (ethics approvals: Erlangen 200_12, 280_14B, and 313_17B, Magdeburg 61/22). Patient characteristics are summarized in Table S1.

For more information, see the Supplemental Information.

To define the landscape of DDR in reconstituting T‐cells after allo‐SCT, we employed a multiparameter flow cytometry approach in CD3^+^ T‐cells at Day +30 post‐transplantation, integrating markers of oxidative DNA damage, BER, DNA replication stress, double‐strand break (DSB) signaling, and exhaustion (Figure 1A). Patients were stratified into 8‐OHdG^lo(w)^, 8‐OHdG^int(ermediate)^, and 8‐OHdG^hi(gh)^ groups according to intracellular 8‐OHdG levels reflecting oxidative DNA damage. This classification was adopted from our previous study.^4^ While it identifies biologically distinct patient subsets, prospective validation in independent cohorts will be required before it can be considered for clinical risk stratification. Consistent with our previous observations,^4^ high oxidative DNA damage was associated with a significantly increased risk of relapse (Figure 1B), underscoring the clinical relevance of oxidative DNA damage in T‐cells during early immune reconstitution after allo‐SCT. Upregulation of BER‐associated proteins, replication stress markers, DSB signaling pathways, and exhaustion markers were found in T‐cells from patients with high oxidative DNA damage (Figure 1C), consistent with enhanced DDR and cellular stress responses to increased DNA damage.

**Figure 1:**
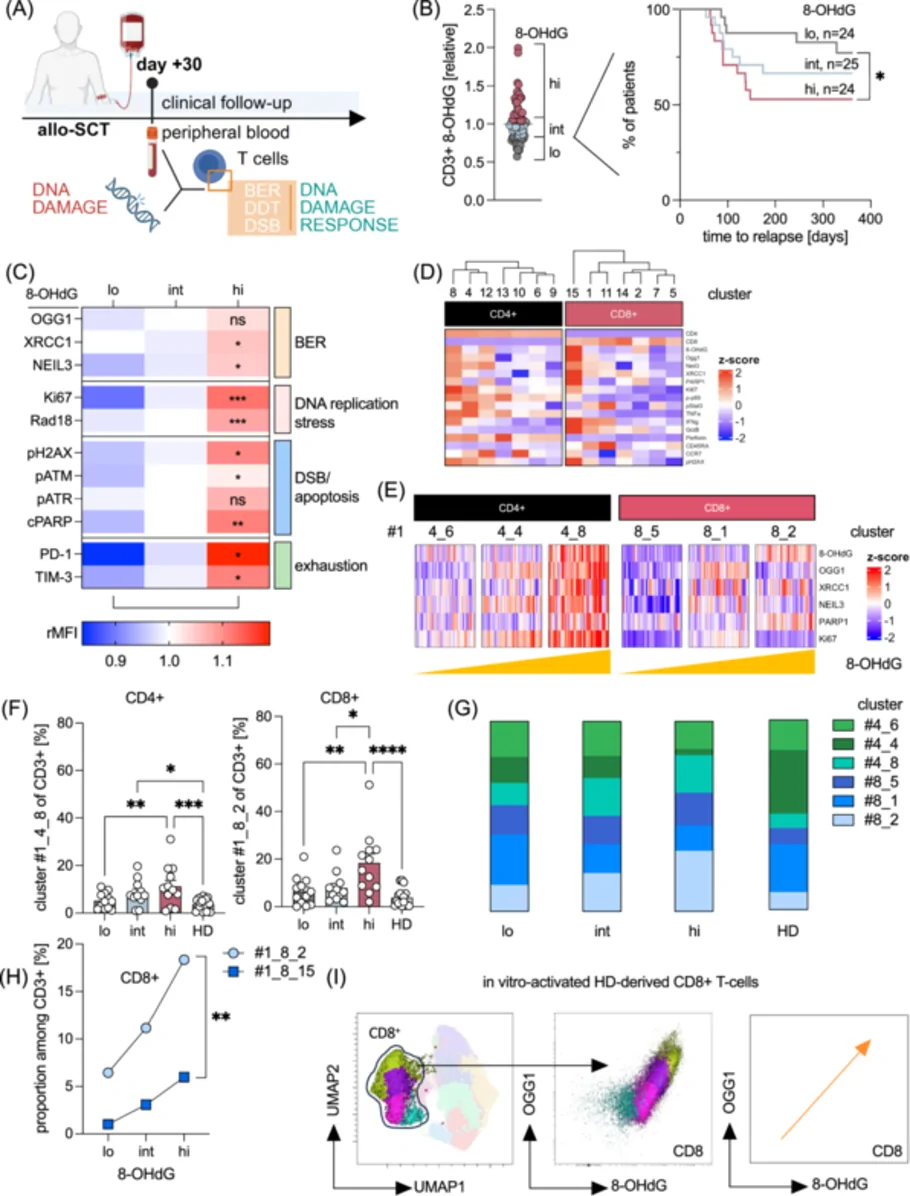
**Oxidative DNA damage reveals maladaptive base excision repair (BER) responses in reconstituting CD8^+^ T‐cells after allogeneic hematopoietic stem cell transplantation (allo‐SCT). (A)** Illustration of sample processing: patient‐derived T‐cells were thawed, and 8‐hydroxy‐2′‐deoxyguanosine (8‐OHdG) formation and DNA damage were semi‐quantified by comprehensive flow cytometry (FACS) panels. Created with BioRender.com (AU29WVL36N). **(B)** Allo‐SCT patients were grouped into low (gray, 0–33 percentile), intermediate (blue, 33–66 percentile), and high (red, >66 percentile) patient groups based on 8‐OHdG level within CD3^+^ T‐cells at Day +30 post‐allo‐SCT of the respective cohort. The relative 8‐OHdG level of CD3^+^ T‐cells at Day +30 after allo‐SCT from both cohorts is shown for 8‐OHdG low (*n* = 23), intermediate (*n* = 25), and high (*n* = 25) patient groups. Time‐to‐relapse for each 8‐OHdG subgroup shown as Kaplan–Meier curve. Events later than Day +360 are censored (*n* = 24–25/group). **(C)** Normalized abundance of key proteins from BER, DNA replication stress, double‐strand break (DSB) repair, and exhaustion are shown in CD3^+^ T‐cells from patients at Day +30 after allo‐SCT. Data of the screening and expansion cohort are plotted as a heatmap (*n* = 24/25/24). Significance was calculated against the 8‐OHdG^lo^ subgroup. **(D)** Flow cytometric data for 8‐OHdG, CD4, CD8, OGG1, NEIL3, XRCC1, PARP1, and pH2AX were used for clustering in a phenograph analysis (#1) of Panel 1 (BER) (*K* = 30) of all samples. CD4^+^ or CD8^+^ clusters are shown, clusters below 5% frequency are not depicted. Heatmap indicates normalized median of each cluster. **(E)** Six clusters of interest from the phenograph analysis #1 were selected based on the highest frequency within CD4^+^ or CD8^+^ T‐cell populations. Normalized expression levels of BER‐related proteins for each patient sample are shown. **(F)** The frequency of high‐damage clusters (#1_4_8, #1_8_2) at Day +30 after allo‐SCT within CD4^+^ and CD8^+^ T‐cells comparing healthy donors (HDs) (*n* = 18/17), 8‐OHdG^lo^ (*n* = 12/12), 8‐OHdG^int^ (*n* = 11/11), and 8‐OHdG^hi^ (*n* = 12/12) patient subgroups. **(G)** The relative abundance of the six clusters of interest within HD, 8‐OHdG^lo/int/hi^ patient subgroups at Day +30 post‐allo‐SCT is shown as normalized to 100%. **(H)** Frequencies of CD8^+^ clusters with the highest 8‐OHdG level at Day +30 post‐allo‐SCT are shown as a percentage of CD3^+^ T‐cells. **(I)** HD‐derived T‐cells (*n* = 3) were cultured for 96 h with activating anti‐CD2/‐CD3/‐CD28 beads and subsequently analyzed by flow cytometry. Phenograph‐derived CD8^+^ clusters are shown in a UMAP plot (left), abundance of 8‐OHdG and OGG1 for all clusters is shown, accordingly (right). Abbreviations: *n* indicates the sample number. *P ≤ 0.05; **P ≤ 0.01; ***P ≤ 0.001; and ****P ≤ 0.0001. DDT, DNA damage tolerance; UMAP, uniform manifold approximation and projection.

Single‐cell clustering of reconstituting T‐cells identified distinct CD4^+^ and CD8^+^ T‐cell states characterized by heterogeneous oxidative DNA damage burden and differential activation of DNA repair, stress‐response, proliferative, and effector programs (Figure 1D). BER represents an early adaptive response to oxidative DNA damage and single‐strand breaks, initiated by DNA glycosylases such as OGG1 and NEIL3 and coordinated by downstream repair proteins including XRCC1 (Figure S1A).^7^ The most frequent CD4^+^ and CD8^+^ T‐cell subpopulations identified by single‐cell clustering are shown in Figure 1E. In CD4^+^ T‐cells, increasing oxidative DNA damage was accompanied by coordinated upregulation of OGG1, NEIL3, and XRCC1, consistent with enhanced BER activity in response to oxidative stress. Notably, the CD4^+^ T‐cell subpopulation with the highest 8‐OHdG burden (i.e., #1_4_8) also exhibited strong Ki67 expression, indicating a highly proliferative state associated with increased replication‐associated vulnerability to oxidative DNA damage. In contrast, CD8^+^ T‐cells with high oxidative DNA damage burden (i.e., #1_8_2) failed to adequately induce BER‐associated proteins despite accumulation of 8‐OHdG. These findings indicate a dissociation between oxidative DNA damage burden and BER induction in a major subset of reconstituting CD8^+^ T‐cells, consistent with a maladaptive response to oxidative stress. The maladaptive CD8^+^ T‐cell subpopulation was markedly enriched in 8‐OHdG^hi^ patients, substantially exceeding the corresponding CD4^+^ T‐cell subpopulation, and accounted for up to 20% of CD3^+^ T‐cells (Figure 1F), indicating that BER‐impaired CD8^+^ T‐cells represent a major component of this prognostically unfavorable T‐cell compartment (Figure 1B). Consistently, increasing oxidative DNA damage was accompanied by a progressive shift in T‐cell composition toward a maladaptive high‐damage CD8^+^ T‐cell state (Figure 1G). While most CD8^+^ T‐cells showed impaired BER induction under oxidative stress, clustering identified a distinct CD8^+^ T‐cell subpopulation (i.e., #1_8_15, Figure S1B) with coordinated upregulation of OGG1, NEIL3, and XRCC1 in parallel with increasing 8‐OHdG levels, in line with a physiological BER response. However, this subpopulation remained significantly underrepresented even under conditions of high oxidative stress (Figure 1H). To determine whether limited BER induction represents a general feature of CD8^+^ T‐cells, we analyzed activated HD‐derived CD8^+^ T‐cells. In contrast to patient‐derived CD8^+^ T‐cells, increasing 8‐OHdG levels were accompanied by proportional OGG1 upregulation (Figure 1I), supporting a physiological linear BER response to oxidative DNA damage and reinforcing the concept of a maladaptive BER‐impaired CD8^+^ T‐cell state after allo‐SCT.

Oxidative DNA lesions are particularly detrimental during proliferation, as they interfere with replication fork progression and trigger replication stress.^8^ We therefore analyzed pathways involved in DNA damage tolerance (DDT) and replication stress adaptation. DDT mechanisms prevent replication fork collapse by enabling replication across damaged DNA templates.^7^ Key components include the E3 ubiquitin ligases RAD18 and RAD5 and DNA polymerase iota (POLI), a translesion polymerase that facilitates lesion bypass. RAD51, RAD51AP1, and BRCA1 mediate homologous recombination (HR) as a complementary pathway for the resolution of replication‐associated DNA lesions and DSBs (Figure 2A). In T‐cells, high levels of 8‐OHdG were associated with higher expression of RAD5, RAD18, and POLI (Figure 2A). Single‐cell clustering identified distinct CD4^+^ and CD8^+^ T‐cell subpopulations characterized by increasing oxidative DNA damage and replication stress. While Figure 2B provides an overview of all identified clusters, Figure 2C highlights the dominant CD4^+^ and CD8^+^ subpopulations arranged according to increasing 8‐OHdG burden. In both lineages, oxidative DNA damage was accompanied by coordinated induction of DDT and HR, indicating preserved replication stress adaptation despite defective BER response observed in a subset of CD8^+^ T‐cells. T‐cell clusters with high DDT and HR activity (i.e., #2_4_7 and #2_8_4) were enriched in 8‐OHdG^hi^ patients (Figure 2D). UMAP (i.e., uniform manifold approximation and projection) revealed cellular regions characterized by high oxidative DNA damage, DDT, and HR activity. Notably, T‐cells derived from patients who subsequently relapsed preferentially localized within these regions, linking oxidative DNA damage and coordinated activation of replication stress‐response networks to inferior outcome (Figure 2E).

**Figure 2:**
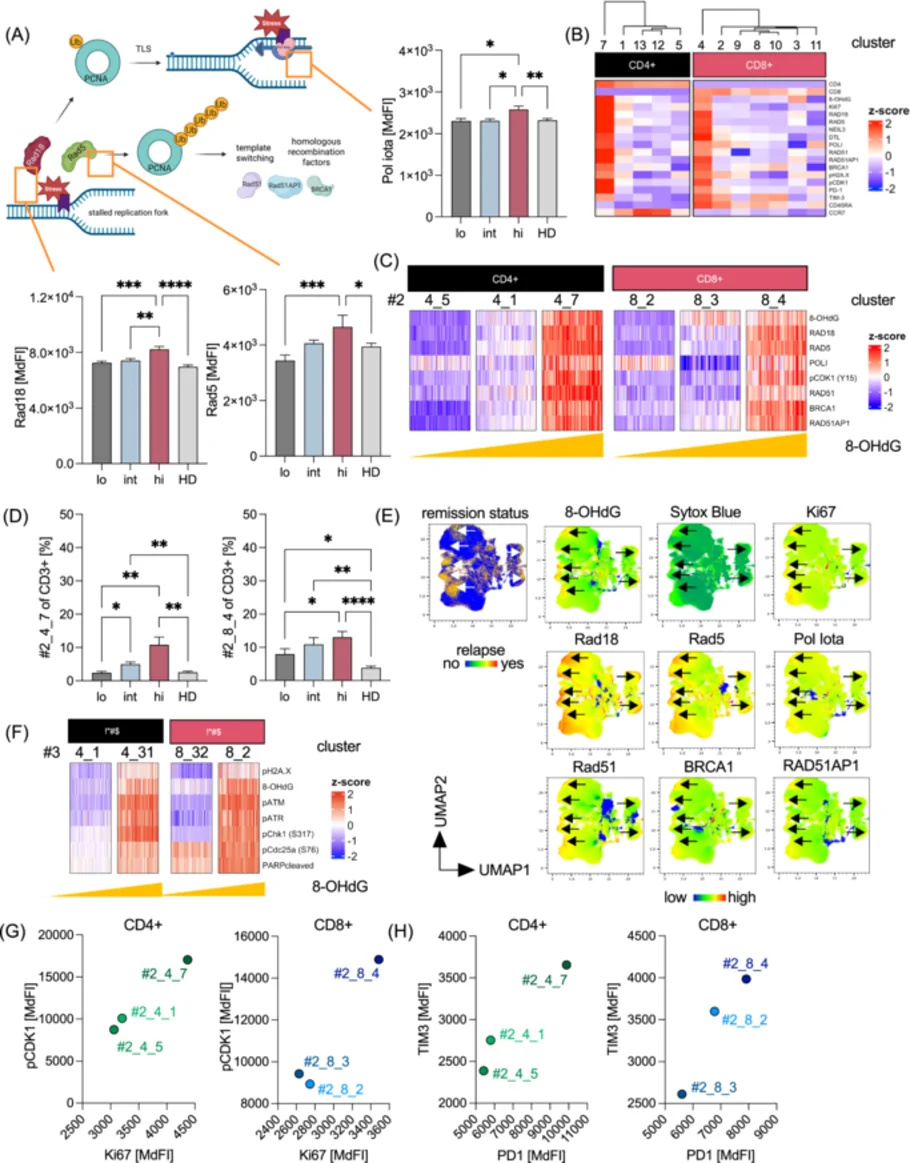
**Oxidative DNA damage induces coordinated activation of DNA damage tolerance, homologous recombination, and checkpoint pathways in both CD4^+^ and CD8^+^ T‐cells after allogeneic hematopoietic stem cell transplantation (allo‐SCT). (A)** Illustration of replication stress‐induced DNA damage tolerance (DDT) pathways. Created with BioRender.com (X29VUG6H3). Median fluorescence index (MdFI) of replication stress‐related proteins in CD3^+^ T‐cells from healthy donor (HD) (*n* = 18/18/16), 8‐OHdG^lo^ (*n* = 12/11/11), 8‐OHdG^int^ (*n* = 11/12/12), and 8‐OHdG^hi^ (*n* = 11/11/11) patient subgroups at Day +30 post‐allo‐SCT. **(B)** Phenograph analysis (#2) of replication stress‐panel including 8‐hydroxy‐2′‐deoxyguanosine (8‐OHdG), CD4, CD8, DTL, NEIL3, pCDK1, pH2AX, DNA polymerase iota (POLI), RAD18, RAD5, RAD51, BRCA1, and RAD51AP1 for clustering (*K* = 100); CD4^+^ or CD8^+^ clusters are shown. Heatmap shows normalized median of each cluster, clusters below 5% frequency are not depicted. **(C)** Six clusters of interest from the phenograph analysis #2 were selected based on the highest frequency within CD4^+^ and CD8^+^ T‐cell population. Normalized expression levels of DDT‐related proteins for each patient sample are shown. **(D)** The frequencies of two selected clusters at Day +30 post‐allo‐SCT are shown for HD (*n* = 17/17), 8‐OHdG^lo^ (*n* = 10/12), 8‐OHdG^int^ (*n* = 12/12), and 8‐OHdG^hi^ (*n* = 12/12) patient subgroups. **(E)** UMAP plot heatmaps show proliferation and DDT‐related markers (right) and occurrence of relapse (left). Arrows indicate populations with high 8‐OHdG levels. **(F)** Phenograph analysis (#3) of double‐strand break (DSB) repair‐panel including 8‐OHdG, CD4, CD8, pH2AX, pATM, pATR, pChk1, pCdc25a, Cdc6, cPARP, and NEIL3 and for clustering (*K* = 100). A comparison of selected low versus high‐damage phenotype clusters is shown for CD4^+^ and CD8^+^ T‐cells depicting the normalized expression of DSB repair‐related proteins for each patient sample. **(G)** Relative abundance of Ki67 and pCDK1 in six selected clusters of interest is plotted based on their median expression level. **(H)** Relative abundance of PD‐1 and TIM3 in six selected clusters of interest is plotted based on their median expression level. Abbreviations: *n* indicates the sample number. *P ≤ 0.05; **P ≤ 0.01; ***P ≤ 0.001; and ****P ≤ 0.0001. UMAP, uniform manifold approximation and projection.

Persistent oxidative DNA damage was associated with activation of DSB‐associated checkpoint pathways. Phosphorylation of H2AX, ATM, ATR, Chk1, and Cdc25a progressively increased with oxidative DNA damage in CD4^+^ and CD8^+^ T‐cells (Figure 2F), indicating coordinated activation of DNA damage sensing and checkpoint signaling. As checkpoint activation delays cell‐cycle progression to allow DNA repair and preserve genome integrity^9^, ^10^ (Figure S1C), highly damaged T‐cell subsets displayed increased pCDK1 expression, consistent with G2/M arrest despite ongoing proliferative activity (Figure 2G). Concurrently, oxidative DNA damage was associated with elevated programmed cell death protein 1 (PD‐1) and T‐cell immunoglobulin and mucin‐domain containing‐3 (TIM‐3) expression, consistent with an exhausted phenotype (Figure 2H).^11^ Thus, oxidative DNA damage appears to impose substantial DDR burden on proliferating T‐cells, linking genome maintenance programs to reduced proliferative fitness and exhaustion. Notably, unlike BER, DDT, HR, checkpoint signaling, and apoptosis remained inducible in both CD4^+^ and CD8^+^ T‐cell subsets.

Analysis of an independent expansion cohort confirmed the coordinated activation of DDR pathways in association with oxidative DNA damage (Figure S2A) and reproduced the presence and enrichment of a BER‐impaired CD8^+^ T‐cell population (Figure S2B). Consistently, DDR pathways were induced across CD4^+^ T‐cell states, whereas CD8^+^ T‐cells displayed greater heterogeneity driven by the BER‐impaired subset (Figure S2C).

As DNA damage and DDR have been implicated in inflammatory signaling,^12^ we explored whether oxidative DNA damage was associated with functional alterations of reconstituting T‐cells. Single‐cell secretome analysis revealed functional differences between 8‐OHdG^lo^ and 8‐OHdG^hi^ patients, which were more pronounced in the CD8^+^ compartment. In particular, CD8^+^ T‐cells from 8‐OHdG^hi^ patients exhibited reduced polyfunctionality and stimulatory activity, accompanied by a shift in cytokine secretion patterns (Figure S2D). Thus, oxidative DNA damage defined a distinct DDR phenotype and also marked graft‐derived T‐cells with impaired polyfunctionality and altered effector programs post‐transplantation. These findings are consistent with emerging evidence linking persistent DNA damage to T‐cell dysfunction. Notably, a recent study demonstrated that oxidative damage at telomeric regions impairs T‐cell fitness and effector functions,^13^ further supporting the concept that genome integrity is critical to effective T‐cell immunity. Although we previously found no evidence that the GvHD prophylaxis regimens used in this study substantially influenced oxidative DNA damage,^4^ future studies should investigate whether contemporary GvHD prophylaxis regimens and different in vivo T‐cell depletion strategies differentially influence oxidative DNA damage and DDR pathways during immune reconstitution after allo‐SCT.

Collectively, these findings suggest that BER represents a selective vulnerability of reconstituting CD8^+^ T‐cells post‐allo‐SCT. While replication stress adaptation, HR, checkpoint signaling, and apoptosis remained inducible, failure to appropriately induce BER was associated with persistent oxidative DNA damage, exhaustion, and impaired T‐cell functionality. Together with our previous work demonstrating restoration of T‐cell fitness upon pharmacologic OGG1 activation,^5^ these data identify BER competence as a potentially targetable determinant of immune reconstitution after allo‐SCT.

## AUTHOR CONTRIBUTIONS

**Tobias Ronny Haage**: Data curation; formal analysis; writing—review and editing; methodology; visualization. **Domenica Saul**: Conceptualization; writing—review and editing; methodology; formal analysis; data curation; visualization. **Romy Böttcher‐Loschinski**: Data curation; formal analysis; visualization; methodology; writing—review and editing. **Martin Böttcher**: Writing—review and editing; project administration; resources. **Simon Völkl**: Writing—review and editing; resources. **Andreas Mackensen**: Writing—review and editing; resources; funding acquisition. **Matthias Leisegang**: Writing—review and editing; methodology. **Maurice Michel**: Methodology; writing—review and editing. **Denise Walther**: Resources; writing—review and editing. **Dimitrios Mougiakakos**: Conceptualization; writing—original draft; writing—review and editing; resources; funding acquisition; formal analysis; data curation; visualization; supervision; methodology.

## CONFLICT OF INTEREST STATEMENT

The authors declare no conflicts of interest.

## FUNDING

T.R.H. was funded by the Research Training Group RTG2408 of the Deutsche Forschungsgemeinschaft (DFG) and the Polycarp‐Leporin‐Program (PLP) of the Otto von Guericke University, Medical Faculty (project PLP25/3). R.B.‐L. was funded by the PLP of the Otto von Guericke University, Medical Faculty (project PLP25/2). A.M. and D.M. were funded by the Deutsche Forschungsgemeinschaft (DFG, German Research Foundation) ‐ Project‐ID 324392634 ‐ TRR 221. D.M. was supported by the European Regional Development Fund (project ZELL‐THEMA). M.M. received funding from the Innovative Medicines Initiative 2 Joint Undertaking (JU) under grant agreement no. 875510. Open Access funding enabled and organized by Projekt DEAL.

## Supporting information


**Supporting Information**.

## Data Availability

Available from the corresponding author upon reasonable request (Dimitrios Mougiakakos, dimitrios.mougiakakos@med.ovgu.de).
