# Supplementary material for: Maladaptive DNA damage responses impair CD8 T‐cell fitness after allogeneic stem cell transplantation
Source: Hemasphere. 2026 Nov 4;10(11):e70525. doi: 10.1002/hem3.70525 (PMC13733361; doi:10.1002/hem3.70525)
Supplement: Supplementary file 1 [file HEM3-10-e70525-s001.pdf]

- Supplementary Information -

Title: Maladaptive DNA damage responses impair CD8 T-cell fitness after allogeneic stem cell transplantation

Running Title: Defective DDR in CD8 T-cells after allo-SCT

Authors/Affiliations: Tobias Ronny Haage[1]*, Domenica Saul[1]*, Romy Böttcher-Loschinski[1], Martin Böttcher[1], Simon Völkl[2], Andreas Mackensen[2], Matthias Leisegang[1], Maurice Michel[3], Denise Walther[1], Dimitrios Mougiakakos[1,4]

[1]Department for Hematology, Oncology, Cell- and Radiotherapy, Otto von Guericke University, 39120 Magdeburg, Germany.

[2]Department of Medicine 5, Hematology and Oncology, Friedrich Alexander University Erlangen-Nuremberg, 91054 Erlangen, Germany.

[3]Department of Oncology and Pathology, Science for Life Laboratory and Center for Molecular Medicine, Karolinska Institute and Karolinska Hospital, 17177 Stockholm, Sweden.

[4]Magdeburg Center for Cell- and Immunotherapy, Otto von Guericke University, 39120 Magdeburg, Germany.

Material and Methods

Flow cytometry

Samples from the screening cohort were fluorescently barcoded using four differently conjugated CD45 antibodies, whereas expansion cohort samples were stained separately. Following viability and surface staining, cells were fixed and permeabilized using the BD Cytofix/Cytoperm™ system combined with BD Cytoperm™ Permeabilization Buffer Plus (BD Biosciences, NJ, USA). Intracellular staining was followed by acquisition on a Cytek NL-3000 full-spectrum flow cytometer. Antibodies are listed in **Supplemental Table 2**.

Secretome analysis

Peripheral blood mononuclear cells were cultured overnight in presence of 10 ng/mL interleukin-2 (Miltenyi Biotec, Germany). CD4 and CD8 T-cells were enriched using EasySep™ Human CD4 or CD8 Positive Selection Kit II (STEMCELL Technologies, Canada) respectively, and stimulated for 3 hours using a Cell Activation Cocktail without brefeldin A (Biolegend, CA, USA). Cells were labeled using Stain Cell Membrane-405 (IsoPlexis, CT, USA), while CD8 T-cells were additionally stained with an anti-CD8 AF647 antibody (Biolegend, CA, USA, clone HIT8a). Following loading onto Single-Cell Secretome Adaptive Immune-Human Chips cells were analyzed using the IsoSpark platform and the IsoSpeak Version 3.0.0 software (IsoPlexis).

Polyfunctionality was assessed as the frequency of cells secreting ≥ 5 analytes simultaneously and by the polyfunctional strength index (PSI), which integrates the frequency of polyfunctional cells and the intensity of cytokine secretion. PSI was determined for the following predefined functional cytokine groups: effector (Granzyme B, IFN-γ, MIP-1α, perforin, TNF-α, TNF-β), chemoattractive (CCL11, IP-10, MIP-1β, RANTES), regulatory (IL-10, IL-13, IL-22, IL-4, sCD137, sCD40L, TGF-β1), and stimulatory (GM-CSF, IL-12, IL-15, IL-2, IL-21, IL-5, IL-7, IL-8, IL-9).

Data analysis

Statistical analyses were performed using GraphPad Prism Version 10.3.0 (GraphPad, CA, USA) and R studio (Posit PBC, MA, USA) with R version 4.2.2 (R Core Team, Austria). Parametric or nonparametric tests were applied according to data distribution. Survival analyses were performed using Kaplan-Meier estimates and log-rank testing. Univariate analyses were conducted using the R package "compareGroups". A p-value <0.05 was considered statistically significant.

Flow cytometry data were analyzed using FlowJo Version 10.8.2 (BD Biosciences) including plugins FlowAI (2.3.2), DownSample (3.3.1), cyCombine (1.0.2), UMAP (4.1.1), and Phenograph (2.5.0). Batch correction was conducted using cyCombine. Parameters used for UMAP and Phenograph analyses are listed in **Supplemental Table 3** and **4**, respectively.

Tables

Table 1: Patients' characteristics.

Variable	Screening cohort [Erlangen]	Extension cohort [Magdeburg]
Number of patients	36	37
Gender		
Female	18 (50%)	19 (51%)
Male	18 (50%)	18 (49%)
Mean age, years (range)	58 (40-71)	58.5 (19-74)
Diagnosis		
AML/MDS	26 (72%)	33 (89%)
Other	10 (28%)	4 (11%)
Donor type		
Haploidentical	1 (3%)	2 (5%)
HLA-identical sibling	7 (19%)	3 (8%)
Unrelated donor	28 (78%)	32 (86%)
Graft source		
PBSC	35 (97%)	36 (97%)
BM	1 (3%)	1 (3%)
HLA compatibility (unrelated donors)		
8/8	21 (75%)	27 (84%)
7/8	7 (25%)	5 (16%)
In vivo T-cell depletion		
ATG	31 (86%)	33 (89%)
PTCy	5 (14%)	4 (11%)
GvHD prophylaxis		
CSA/MMF	19 (53%)	37 (100%)
CSA/MTX	12 (33%)	0 (0%)
Other	5 (14%)	0 (0%)

Abbreviations: AML, acute myeloid leukemia; ATG, anti-thymocyte globulin; BM, bone marrow; CSA, cyclosporine A; GvHD, graft-versus-host disease; HLA, human leukocyte antigen; MDS, myelodysplastic syndrome; MMF, mycophenolate mofetil; MTX, methotrexate; PBSC, peripheral blood stem cells; PTCy, post-transplant cyclophosphamide.

Table 2: List of antibodies.

Antigen	Fluorochrome	Clone	Isotype	Identifier	Company
8-OHdG	FITC	15A3	Mouse IgG2b, κ	ab183393	abcam
ATM (S1981)	CF405M	polyclonal	rabbit IgG	BYT-ORB304688-CF405M	Biozol
ATR (T1989)	unconjugated	EPR21991	rabbit IgG	ab223258	abcam
	PE-Cy7 Kit			ab102903	abcam
BRCA1	PE-Cy5.5	polyclonal	rabbit IgG	BYT-ORB917297	Biozol

CCR7	BV650	G043H7	Mouse IgG2a, κ	353234	Biolegend
CD3	APC-Fire810	SK7	Mouse IgG1, κ	344858	Biolegend
CD4	BV605	SK3	Mouse IgG1, κ	562658	BD Biosciences
	PE-Vio770	VIT4	Mouse IgG2aκ	130-113-227	Miltenyi
CD8	BV711	SK1	Mouse IgG1, κ	563677	BD Biosciences
CD45	AmCyan	2D1	Mouse IgG1, κ	339192	BD Biosciences
	NF Blue 585	KI30	Mouse IgG1, κ	H029T03B04	Thermo Fisher
	NF Yellow 730	KI30	Mouse IgG1, κ	H029T03Y07	Thermo Fisher
	PE-Fire640	KI30	Mouse IgG1, κ	304074	Biolegend
	Qdot800	KI30	Mouse IgG1, κ	Q10156	Thermo Fisher
CD45RA	BV570	HI100	Mouse IgG2bκ	304132	Biolegend
CD70	PE	113-16	Mouse IgG1, κ	355104	Biolegend
CD127 (IL7Ra)	BV510	A019D5	Mouse IgG1, κ	351332	Biolegend
Cdc6	AF532	OTI1A4	Mouse IgG1	NBP2-70383AF532	Novus biologicals
Cdc25a (S76)	PE-Cy5.5	polyclonal	rabbit IgG	BYT-ORB904891	Biozol
CDK1 (Y15)	AF680	polyclonal	rabbit IgG	BSS-BS-3092R-A680	Biozol
Chk1 (S317)	PE	Chk1S317-G1	rabbit IgG	MA5-27984	Thermo Fisher
DNA	SYTOX Blue			S34857	Invitrogen
DTL	APC	polyclonal	rabbit IgG	USB-034834-APC	Biozol
GrzB	PE-CF594	GB11	mouse IgG1, κ	562462	BD Biosciences
H2A.X (Ser139)	PE	CR55T33	mouse IgG1, κ	12-9865-42	Thermo Fisher
H2A.X (Ser139)	PerCP-eFluor710	CR55T33	mouse IgG1, κ	46-9865-42	Thermo Fisher
IFNγ	BV750	4S.B3	mouse IgG1, κ	502550	Biolegend
Ki-67	BV421	Ki-67	Mouse IgG1, κ	350506	Biolegend
KLRG1	AF700	SA231A2	Mouse IgG2a, κ	367730	Biolegend
NEIL3	AF750	polyclonal	rabbit IgG	NB100-441AF750	Novus biologicals
Ogg1	PE-Cy5.5	2B4	Mouse IgG2b	NBP2-52724PECY55	Novus biologicals
p53 (S37)	APC	REA424	human IgG1	130-106-556	Miltenyi
p65 (S529)	PerCP-eFluor710	B33B4WP	mouse IgG2a, κ	46-9863-42	Thermo Fisher
PARP, cleaved	PE-CF594	F21-852	mouse IgG1, κ	564130	BD Biosciences
PARP1	CF405M	polyclonal	rabbit IgG	BYT-ORB213531-CF405M	Biozol
PD-1	BV750	EH12.2H7	mouse IgG1, κ	329966	Biolegend
Perforin	PerCP	B-D48	Mouse IgG1, κ	BYT-ORB308541	Biozol
POLI	CF405M	polyclonal	rabbit IgG	BYT-ORB156613-CF405M	Biozol
RAD18	AF532	polyclonal	rabbit IgG	NBP2-98850AF532	Novus biologicals
RAD51	PerCP	RAD51/2701	mouse IgG2b	NBP2-79895PCP	Novus biologicals
RAD51AP1	PE	polyclonal	rabbit IgG	BYT-ORB489729	Biozol

SHPRH/RAD5	PE-Cy7	OTI5G8	mouse IgG2bκ	NBP2-74180PECY7	Novus biologicals
Stat3(pY705)	AF647	Clone 4/P-STAT3 (RUO)	Mouse IgG2a, κ	557815	BD Biosciences
TIM-3	BV785	F38-2E2	mouse IgG1, κ	345032	Biolegend
TNFa	BV605	MAb11	mouse IgG1, κ	502936	Biolegend
Viability	Live-or-Dye 665/685			BOT-32013	Biozol
XRCC1	AF700	OTI2F8	mouse IgG1	NBP2-74891AF700	Novus biologicals

Table 3: UMAP parameters used for single-cell clustering and visualization.

**Screening cohort**			**Extension cohort**
panel 1	panel 2	panel 3	selected parameters
8-OHdG	8-OHdG	8-OHdG	8-OHdG
CCR7	BRCA1	CCR7	CD4
CD4	CCR7	CD4	CD70
CD45RA	CD4	CD45RA	CD8
CD8	CD45RA	CD8	cPARP
GrzB	CD8	Cdc6	NEIL3
IFNγ	DTL	cPARP	OGG1
Ki-67	Ki-67	IL7Ra	pATM
NEIL3	NEIL3	Ki-67	pATR
OGG1	pCDK1	KLRG1	pH2AX
PARP1	PD-1	NEIL3	RAD18
perforin	pH2AX	pATM	XRCC1
pH2AX	PoI iota (POLI)	pATR	
p65(S529)	RAD18	pCdc25a	
pSTAT3	RAD5	pChk1	
TNFa	RAD51	pH2AX	
XRCC1	RAD51AP1	p53(S37)	
	Tim3		

Note: Uniform Manifold Approximation and Projection (UMAP) was performed using Euclidean distance with 15 nearest neighbors, a minimum distance of 0.5 and two embedding dimensions.

Table 4: Phenograph clustering parameters.

**Screening cohort**			**Extension cohort**
panel 1	panel 2	panel 3	selected parameters
8-OHdG	8-OHdG	8-OHdG	8-OHdG
CD4	BRCA1	CD4	CD4
CD8	CD4	CD8	CD70
NEIL3	CD8	Cdc6	CD8
OGG1	DTL	cPARP	cPARP
PARP1	NEIL3	NEIL3	NEIL3
pH2AX	pCDK1	pATM	OGG1
XRCC1	pH2AX	pATR	pATM
	PoI iota	pCDC25a	pATR
	RAD18	pChk1	pH2AX
	RAD5	pH2AX	RAD18

	RAD51		XRCC1
	RAD51AP1		

Figures

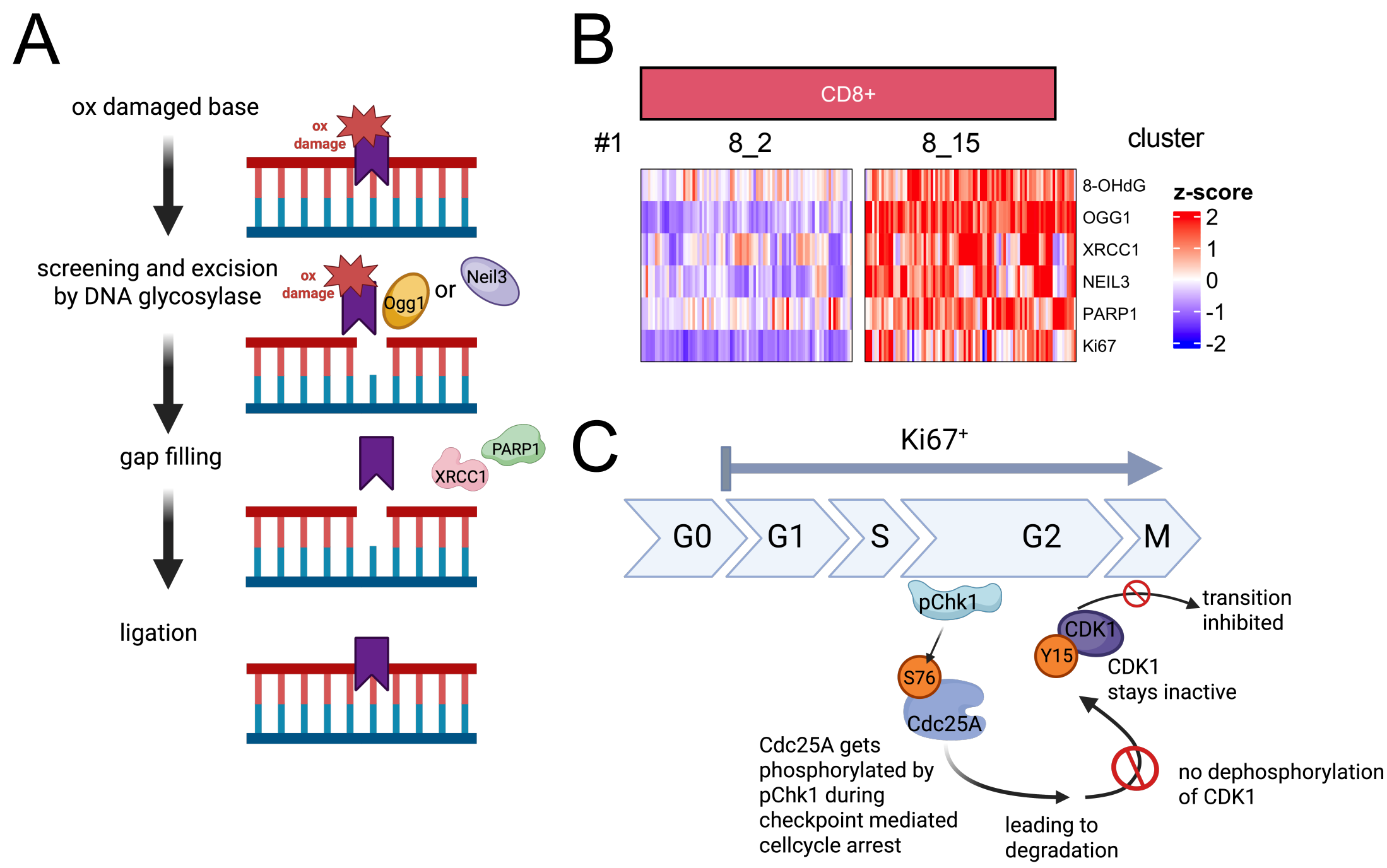

Supplemental Figure 1. (A) Schematic overview of the base excision repair (BER) pathway. Oxidative DNA lesions are recognized and excised by the DNA glycosylases 8-oxoguanine DNA glycosylase 1 (OGG1) and endonuclease VIII-like protein 3 (NEIL3), thereby initiating BER. Subsequent processing of abasic sites is coordinated by X-ray repair cross-complementing protein 1 (XRCC1) and poly(ADP-ribose) polymerase 1 (PARP1), followed by gap filling and DNA ligation. Created with *BioRender.com* (LU29VU9YKM). **(B)** Representative heatmap of the two predominant CD8+ T-cell clusters identified by PhenoGraph analysis. Cluster 8_15 exhibits coordinated upregulation of BER-associated proteins, whereas cluster 8_2 is characterized by high oxidative DNA damage despite limited induction of BER components, consistent with an impaired BER response. **(C)** Schematic illustration of checkpoint-mediated G2/M arrest. Activation of the ATR-Chk1 pathway promotes phosphorylation and degradation of Cdc25A, thereby preventing activation of cyclin-dependent kinase 1 (CDK1). Consequently, cells retain Ki-67 expression but fail to progress into mitosis, consistent with checkpoint-mediated G2/M arrest. Created with *BioRender.com* (license number: TY29VUAGK3).

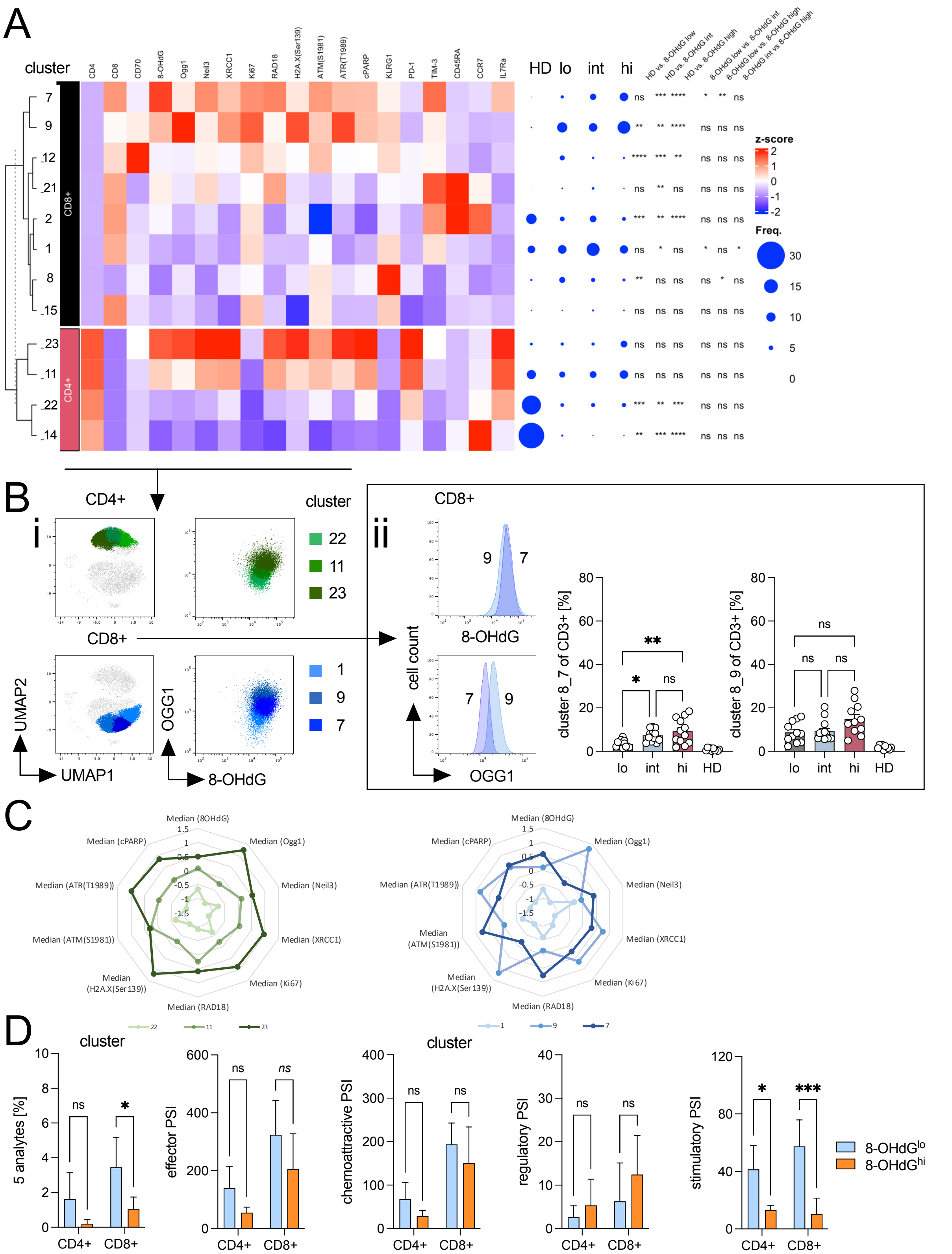

Supplemental Figure 2: (A) PBMCs from healthy donors (HD, n=10) and allo-SCT recipients (n=37) were analyzed *ex vivo* by multiparameter flow cytometry. PhenoGraph clustering was performed using CD4, CD8, 8-OHdG, 8-oxoguanine DNA glycosylase 1 (OGG1), endonuclease VIII-like protein 3 (NEIL3), X-ray repair cross-complementing protein 1 (XRCC1), RAD18, pH2AX, pATM, pATR, cPARP, and CD70. Only CD4+ and CD8+ T-cell clusters with a frequency ≥5% are shown. The heatmap

displays normalized median marker expression within each cluster. Dot size represents cluster frequency in HD, 8-OHdG^{lo}, 8-OHdG^{int}, and 8-OHdG^{hi} patient groups. Asterisks indicate significant differences in cluster frequencies between groups. **(B)** UMAP visualization of selected CD4+ (green) and CD8+ (blue) T-cell clusters identified by PhenoGraph analysis (i). Representative overlays illustrate the relationship between 8-OHdG and OGG1 expression within CD8+ T-cell clusters. Bar graphs show the frequencies of the BER-impaired (cluster 8_7) and BER-competent (cluster 8_9) CD8+ T-cell populations in HD and the respective patient groups (ii). **(C)** Radar plots comparing normalized expression of BER-, DNA replication stress-, and DSB repair-associated markers in selected CD4+ and CD8+ T-cell clusters. **(D)** Single-cell secretome analysis of restimulated CD4+ and CD8+ T cells from 8-OHdG^{lo} and 8-OHdG^{hi} allo-SCT recipients (each n=4). Shown are the frequency of highly polyfunctional cells (≥5 analytes) and the polyfunctional strength index (PSI) for effector, chemoattractive, regulatory, and stimulatory cytokine programs. Error bars represent the standard error mean. Abbreviations: 'n' indicates the sample number. *, p≤0.05; **, p≤0.01; ***, p≤0.001, ****, p≤0.0001.
